# Neuron Contact Detection Based on Pipette Precise Positioning for Robotic Brain-Slice Patch Clamps

**DOI:** 10.3390/s23198144

**Published:** 2023-09-28

**Authors:** Ke Li, Huiying Gong, Jinyu Qiu, Ruimin Li, Qili Zhao, Xin Zhao, Mingzhu Sun

**Affiliations:** 1 Institute of Robotics and Automatic Information System, Tianjin Key Laboratory of Intelligent Robotics, Nankai University, Tianjin 300350, China; like@mail.nankai.edu.cn (K.L.); huiyinggong@mail.nankai.edu.cn (H.G.); qiujinyu@mail.nankai.edu.cn (J.Q.); lrumin@mail.nankai.edu.cn (R.L.); zhaoqili@nankai.edu.cn (Q.Z.); zhaoxin@nankai.edu.cn (X.Z.); 2 Institute of Intelligence Technology and Robotic Systems, Shenzhen Research Institute of Nankai University, Shenzhen 518083, China

**Keywords:** neuron contact, pipette precise positioning, robotic patch clamp

## Abstract

A patch clamp is the “gold standard” method for studying ion-channel biophysics and pharmacology. Due to the complexity of the operation and the heavy reliance on experimenter experience, more and more researchers are focusing on patch-clamp automation. The existing automated patch-clamp system focuses on the process of completing the experiment; the detection method in each step is relatively simple, and the robustness of the complex brain film environment is lacking, which will increase the detection error in the microscopic environment, affecting the success rate of the automated patch clamp. To address these problems, we propose a method that is suitable for the contact between pipette tips and neuronal cells in automated patch-clamp systems. It mainly includes two key steps: precise positioning of pipettes and contact judgment. First, to obtain the precise coordinates of the tip of the pipette, we use the Mixture of Gaussian (MOG) algorithm for motion detection to focus on the tip area under the microscope. We use the object detection model to eliminate the encirclement frame of the pipette tip to reduce the influence of different shaped tips, and then use the sweeping line algorithm to accurately locate the pipette tip. We also use the object detection model to obtain a three-dimensional bounding frame of neuronal cells. When the microscope focuses on the maximum plane of the cell, which is the height in the middle of the enclosing frame, we detect the focus of the tip of the pipette to determine whether the contact between the tip and the cell is successful, because the cell and the pipette will be at the same height at this time. We propose a multitasking network CU-net that can judge the focus of pipette tips in complex contexts. Finally, we design an automated contact sensing process in combination with resistance constraints and apply it to our automated patch-clamp system. The experimental results show that our method can increase the success rate of pipette contact with cells in patch-clamp experiments.

## 1. Introduction

The 21st century is known as the age of neuroscience. More and more scientists are attempting to study the electrophysiology signaling mechanisms of brain activity at the cellular level. Patch-clamp techniques directly measure and record the ion-channel electrical signals at the molecular level; hence, the patch clamp is known as the “gold standard” for studying ion-channel biophysics and pharmacology [1]. In patch-clamp experiments, operators need to perform a series of complex operations within a limited time, including pipette motion control, pipette pressure regulation, cell detection, signal measurement, and so on. Each operation influences the success rate of the experiment. Patch-clamp experiments heavily depend on the professional quality and operational experience of the operators, which is a huge test of their physical and mental capabilities. Automatic or robotic patch clamps can help reduce the operational intensity for operators and improve the success rate of experiments, which promotes the rapid development of brain science research [2].

Recently, many automatic or robotic patch-clamp systems have been developed for brain slices. Kolb et al. built a robotic system known as “PatcherBot” that can perform sequential patch-clamp recordings. The system has been applied to many studies on single-cell measurement [3]. Holst et al. designed and developed an automatic patch-clamp system capable of automating pipette filling, pipette positioning, neuron detection, membrane rupture, and stimulation transmission, which ensures control over record quality, thereby realizing cell type characterization in vivo [4].

Automatic brain-slice patch-clamp experiments are usually performed under a 40× water immersion objective of the microscope. In these experiments, a pipette filled with electrode fluid first comes into contact with the target neuron. Then, negative pressure is applied to the pipette so that part of the cell membrane is aspirated into the pipette to form a giga-seal between the cell surface and the inner surface of the pipette. Next, the cell membrane is broken with high negative pressure pulses to form a whole-cell state. Finally, the electrophysiological signals are recorded. Among them, the contact between the pipette and the neuron is the first and most important step. If the pipette does not have sufficient contact with the cell, it will cause the failure of the cell seal or membrane break-in, decreasing the success rate of the experiment. If the electrode presses the cell too tightly, it will cause cell death and the failure of the electrophysiological signal recording. Therefore, it is necessary to accurately control the contact between the pipette tip and the neuron surface, which will greatly improve the success rates of the subsequent steps of the patch-clamp experiments.

The contact between the pipette and neuron mainly includes two key parts: first, the pipette accurately moves to the place above the target neuron, aligning the electrode tip with the target neuron on a plane. Then, the pipette continuously moves down to approach the target neuron surface until contact is complete. In previous studies, researchers have achieved target neuron positioning in 3D [5,6]. The 3D positioning of the pipette in a complex background of brain slices is also important for the experiments [7,8]. The pipette could quickly approach the neurons after electrode positioning. Therefore, in this paper, we focus on the precise positioning of the pipette and the contact detection between the pipette and the neuron.

In automatic micromanipulation, the manipulation tool is usually focused based on the clarity evaluation indicators [9,10], and then its tip is detected using image processing methods [11], achieving 3D positioning of the manipulation tool. However, in patch-clamp experiments, the pipette is assembled on the micromanipulator at an angle. Different parts of the pipette are located in different focal planes of the microscope. The traditional clarity evaluation indicators cannot distinguish focal planes with different depths under a 40× objective lens with a small field of view and short depth of field. The plane positioning methods of the pipette include image intensity analysis [12], template matching [13], line intersection detection [14], and so on. Wang et al. proposed a convolutional neural network (CNN) method for synchronous depth estimation and object localization in cell manipulation [15]. However, this method requires both the focused and defocused images of the pipette as input, limiting its application in patch-clamp experiments. Li et al. proposed a framework to build a monocular visual-tactile sensor for robotic manipulation tasks [16]. However, in our experiment, the resistance value of the pipette is also one of the criteria for the success of the experiment. Therefore, additional sensors cannot be added for detection.

**Figure 1:**
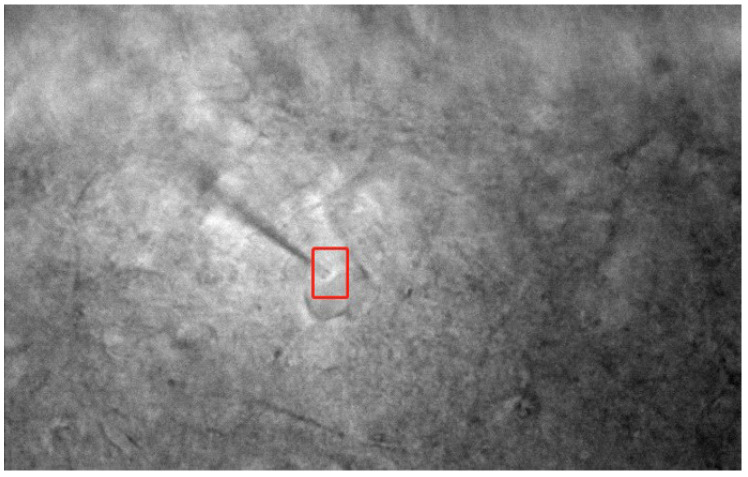
Pipette makes contact with neuronal cells to produce dents.

**Figure 2:**
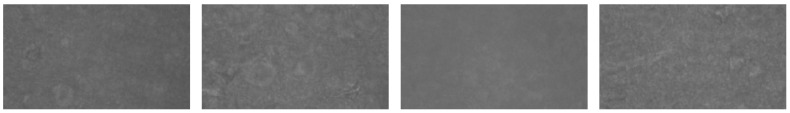
Slice images of brain slices in a patch-clamp experimental environment.

For contact detection, the operators usually determine the contact between the pipette and the neuron through visual observation, i.e., whether the cell has been dented by the tip of the pipette, as shown in Figure 1. The white near the tip of the pipette is produced by depression. However, in the complex environment of the brain slice, blood vessels or other tissues are distributed around neurons, resulting in unclear cell contours and few features in the microscopic image, as shown in Figure 2. It is difficult even for the operators to distinguish the dent of the neuron and the normal shadows due to the influence of DIC imaging. In addition, the resistance of the pipette, which is measured after the pipette enters the extracellular fluid and before it contacts the neuron surface, is usually called bath impedance. This resistance value indicates the distance relationship between the pipette and the neuron, so it is an important indicator in patch-clamp experiments. In our previous work, a non-contact cell measurement method was established using the pipette resistance to obtain the surface shape of neurons [17]. In early automatic patch-clamp systems, the pipette resistance was also used to determine the stage of the experiment [18,19]. However, the movement of pipettes exhibits significant randomness in the brain-slice environment, due to the lack of visual feedback in these systems. The increase in resistance can only infer the contact between the pipette and some objects in brain tissue, but it cannot determine whether these objects are neurons or other impurities, such as blood vessels, leading to an extremely low success rate of patch-clamp experiments [20]. Recently, visual detection has been introduced into the automatic patch-clamp process [12,21]. However, there are still a large number of misjudgments in contact detection, since the visual methods only focus on the detection and localization of the pipette and neuron and have not combined this information with the resistance indicators.

In this study, we propose a neuron contact detection method by combining the visual positioning of the pipette tip and the analysis of the pipette resistance. We first obtain the precise position of the pipette tip so that the pipette tip can move to the position immediately above the target neuron. Subsequently, we convert the contact detection between the pipette and the neuron into the focus detection of the pipette tip, according to the experimental strategy used by neurobiology experts. Finally, contact detection is achieved regarding the pipette resistance as a constraint.

The main contributions of this study are as follows:

(1) A pipette-tip positioning method is proposed for the tilting electrode in a complex brain-slice background. The visual focusing is first converted into motion detection according to the imaging characteristics of the tilted electrode, realizing pipette-tip region focusing. Then, the deep learning-based object detection and scanning line analysis are integrated to achieve the precise positioning of the pipette tip in its bounding box.
(2) A visual contact detection method is proposed based on neuron and pipette focusing. A multi-task convolutional network is designed to determine whether the pipette tip is focused.
(3) An automatic process for contact detection is designed, which regards the pipette resistance as a constraint and automatically determines whether the pipette and the target neuron have successfully made contact.

## 2. Methods

### 2.1. Pipette Precise Positioning

#### 2.1.1. Pipette Focusing Based on Motion Analysis

**Figure 3:**
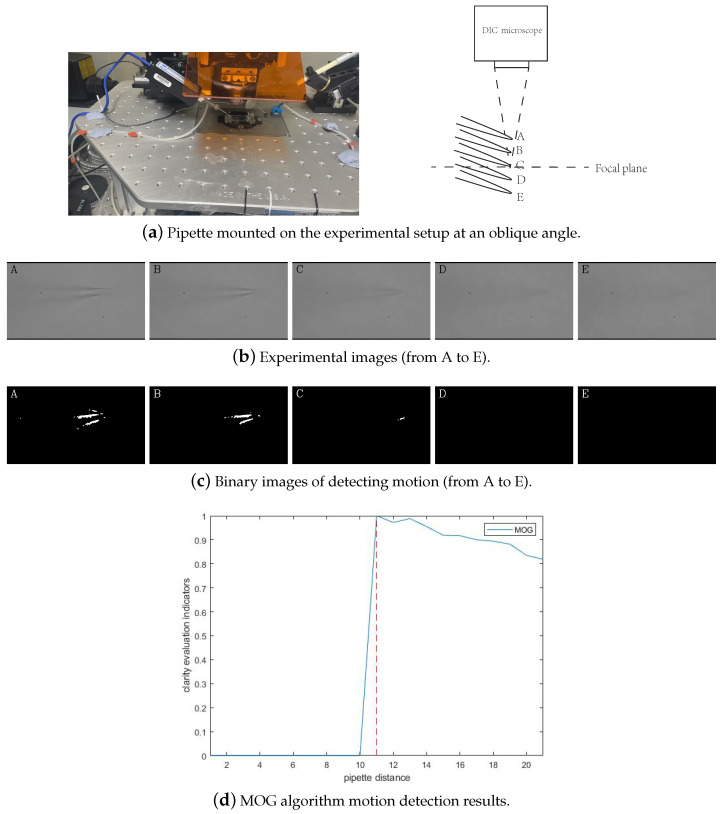
MOG algorithm motion detection process.

The goal of pipette deep positioning is to focus on the tip region of the pipette so that we can subsequently obtain the plane positioning of the pipette tip in the microscopic image. As shown in Figure 3a, the pipette is assembled on the micromanipulator at an angle. Figure 3b shows the microscopic images when the pipette moves in the depth direction. When the pipette is visible in the microscopic images (Figure 3b (A–C)), there is always a part of the pipette that is focused. We cannot obtain a completely clear pipette image during focusing, which makes the traditional autofocusing strategies based on the clarity evaluation indicator infeasible. When the pipette is completely defocused, the pipette tip is located above the focus plane of the microscope. At this time, the movement of the pipette will not change the microscopic image (Figure 3b (D,E)). In this paper, we propose a pipette-focusing method based on motion detection, which converts the focusing of the pipette tip into motion detection in the microscopic image sequence. First, we put the pipette in a near-defocusing state. Then we increase the distance between the pipette and the microscope continuously and detect the motion in the image sequence. When the movement of the pipette is detected, the pipette tip is focused.

In this paper, we use the MOG algorithm [22,23] for motion detection. This algorithm uses a mixture of multiple Gaussian functions to model the background pixels and weights each Gaussian function using the existence time of pixels in the time series. The MOG-based motion detection method not only has strong adaptability to complex scenes but also adjusts the background model through automatically calculated model parameters. The Gaussian distribution modeling for pixel P is expressed as follows:

(1) $$P\left(I\left(x,y,t\right)\right)=\eta \left(x,\mu_{t},\sigma_{t}\right)=\frac{1}{\sqrt{2\pi \sigma_{t}}}e^{\frac{{\left(x-\mu_{t}\right)}^{2}}{2\sigma_{t}^{2}}}$$

The image is divided into 3∼5 Gaussian models. For each pixel, if the distance between the grayscale of the pixel and any Gaussian model is greater than 2 times its standard deviation, it is a foreground pixel or belongs to the moving object; otherwise, it is a background pixel. All the background pixels are combined to form the background image $B_{n}$. The foreground of the current image can be represented as the absolute value of the pixel subtraction between the current image and the background image. Set the current image to $f_{n}$, and the foreground image $D_{n}$ can be expressed as:

(2) $$D_{n}\left(x,y\right)=\left|B\left(x,y\right)-f_{n}\left(x,y\right)\right|$$

where, B(x,y) and $f_{n}\left(x,y\right)$ represent the corresponding pixels in the background and current frames, respectively. Set a threshold *T* and obtain the binary image $R_{n}$ of the foreground image $D_{n}$:

(3) $$R_{n}\left(x,y\right)=\left\{\begin{array}{ccc} 255 & \; & D_{n}\left(x,y\right)>T\\ 0 & \; & else \end{array}\right.$$

In the binary images, the point with a grayscale value of 255 represents the pixel from the moving object, and the point with a grayscale value of 0 represents a background pixel, as shown in Figure 3c. We construct a new clarity evaluation indicator based on the binary image. As shown in Figure 3d, the curve of the clarity indicator has a step when the pipette tip approaches the focusing plane of the microscope and the red dash line is where the pipette tip is focused. This position corresponds to the focal plane of the microscope, completing the focusing of the pipette.

#### 2.1.2. Pipette Plane Positioning Based on Scanning Line

The pipettes on the patch-clamp system are made using a micropipette puller, which melts the borosilicate glass tubes using a heated platinum sheet and pulls them into the micropipette for cell micromanipulation. However, the platinum sheet is consumable and will have slight deviations after a period of use, resulting in different shapes of the pipettes, even if the parameters of the micropipette puller are the same. In addition, different experiments need different resistance values of the pipettes, which leads to different shapes of the pipettes [24]. Therefore, we combine the convolutional neural network (CNN) and image intensity analysis for precise and robust positioning of the pipettes.

The object detection algorithms based on deep learning automatically extract image features based on the convolutional neural network structure. The algorithms convert the input into high-dimensional features, meeting the recognition and classification requirements of complex environments and objects. At present, object detection algorithms based on deep learning can be divided into two categories: two-stage detection algorithms and one-stage detection algorithms. Fast R-CNN [25] is the most common two-stage detection algorithm. In its first stage, a region proposal network (RPN) is used to distinguish the foreground and background and obtain the proposal regions of the objects by introducing a set of bounding boxes. In its second stage, a regression network is used to classify and fine-tune different objects. However, two-stage detection algorithms only extract and classify the features from regions of interest (ROI), ignoring the spatial information of local targets in the whole image. So, one-stage detection algorithms are proposed. YOLO series algorithms [26] are the most famous one-stage detection algorithms, and they directly use regression methods for object classification and bounding box prediction without region proposals. YOLO has well-established applications in the field of cell detection [27]. Compared with two-stage detection algorithms, one-stage detection algorithms save the step of generating the initial bounding box, increasing the detection efficiency by one order of magnitude. The latest YOLO algorithms are YOLOv7 [26] and YOLOv8 [28]. The test results show that the YOLO algorithms have much higher accuracy than the Fast R-CNN algorithm. Compared to YOLOv8, YOLOv7 has a faster detection speed and adequately meets the real-time requirements. Therefore, we utilize YOLOv7 for pipette detection.

**Figure 4:**
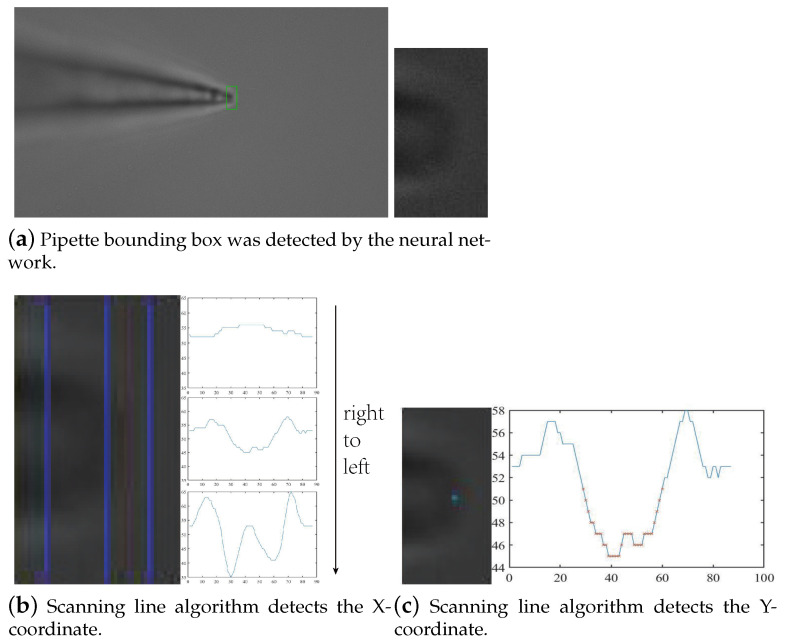
Pipette plane positioning.

We only obtain the bounding box of the pipette using the object detection algorithm, as shown in Figure 4a. The green bounding box is the result of the object detection model. It would cause significant position errors if the center of the bounding box was simply regarded as the coordinates of the pipette tip. Since we have already obtained the ROI of the pipette tip, we can obtain the accurate coordinates of the tip within the ROI image. In this image, there is a significant difference between the pipette and the background and there are small impurities in the background. We employ the scanning line analysis method for the precise positioning of the pipette tip.

Since the pipette is displayed horizontally in the microscope, we first move the vertical scanning line in the ROI image to obtain the X-coordinate of the pipette tip, then perform intensity analysis on the corresponding vertical scanning to obtain the Y-coordinate. As shown in Figure 4b, the scanning line moves horizontally from right to left, and the grayscale values of each column in the image are recorded. The region located to the right of the pipette tip is the background region, and the grayscale changes smoothly on the scan line. Subsequently, in the pipette-tip region, there is a grayscale peak valley and two peaks on the scan line due to the black parts on the tip. Finally, in the region located to the left of the pipette tip, the black tube with internal electrode fluid can be seen, resulting in more peaks on the scanning line. We obtain the minimum grayscale value on each scanning line and set the percentile of the grayscale value as the reference value. When the minimum grayscale value on the scanning line is less than 80% of the reference value, the position of the scanning line is determined as the X-coordinate of the pipette tip. Further, we collect the pixels whose grayscale values are less than 80% of the reference value on this scanning line and calculate the average of the Y-coordinates of all these pixels as the Y-coordinate of the pipette tip, as shown in Figure 4c. The red symbols are pixels that meet the requirements.

### 2.2. Visual Contact Detection between Pipette and Neuron

**Figure 5:**
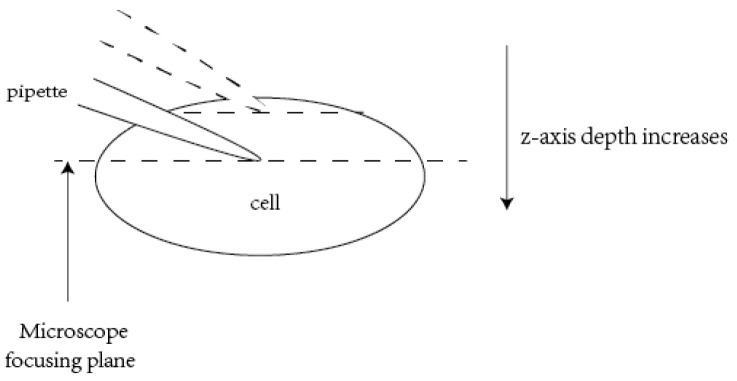
Schematic diagram of defocus prediction.

As shown in Figure 1, the dents of the neurons made by the pipette in the patch-clamp experiment are not obvious, exhibiting fewer features. It is considerably difficult to detect the dents using image processing methods due to the lack of effective visual information. In this study, the contact detection between two types of objects, the pipette and the neuron, is converted into the focusing of the single objects. We first focus the microscope on the plane with the largest cell area and then lower the pipette to bring it into focus, ensuring that the two have sufficient contact, as shown in Figure 5.

#### 2.2.1. Neuron Focusing Based on 3D Positioning

**Figure 6:**
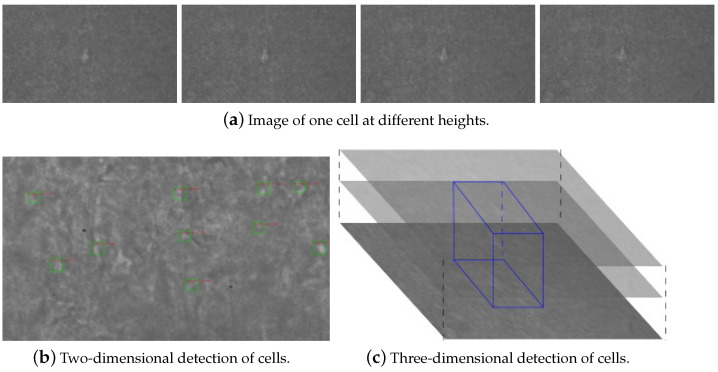
Depth-based three-dimensional detection of neuronal cells.

In electrophysiological experiments, even the best operators cannot find suitable neurons in complex environments quickly and accurately due to the sulcus and gyrus in brain slices. The same neuron will show different shapes at different focal depths, as shown in Figure 6a. Neuron detection based on a single microscopic image will lose the visual information of other neurons at adjacent depths. Thus, the operators not only observe the current microscopic image but also refer to the microscopic images near the focus plane through continuous focusing, that is, they use the 3D characteristics to select neurons.

In this study, we detect the 3D information of the neurons inspired by the manual experimental process. Specifically, the bounding boxes of the neurons are obtained using the object detection model in the microscope images of adjacent focus planes. Then, the bounding boxes at different depths are fused to obtain a 3D bounding box of the neuronal cells. Finally, the optimal focus positions of the neurons are obtained.

We first collect 50 brain-slice images in the depth direction with a step of 1 μm. We then utilize an object detection model to locate all the neurons in all the images and obtain the bounding boxes of the neurons in each image, as shown in Figure 6b. The green box is the bounding box for cell detection and the red is the score. Moreover, we collect the bounding boxes with the same plane position and different depths and use the union operation to expand the area of the bounding boxes. The following strategies are employed to fuse a series of plane bounding boxes to a 3D bounding box: (1) For the same neuron, its depth interval should be less than 2 μm; otherwise, the bounding boxes do not belong to the same neuron, even if they are at the same location; (2) The thickness of the neuron should be greater than 3 μm, which means that there should be more than 3 sequential images of the neuron at the same location; (3) Bounding boxes with an overlap of 60% can be regarded as belonging to the same neuron.

Strategy 1 ensures that the 3D bounding box contains only one neuron, avoiding the incorrect identification of multiple neurons at different depths in the same position as one neuron. Strategy 2 ensures that the 3D bounding box contains real neurons, rather than impurities with similar features to neurons. Strategy 3 ensures the fusing of adjacent bounding boxes. Figure 6c shows the 3D bounding box. We use the middle of the 3D bounding box as the optimal focus position, as the neuron has the maximum area in the microscopic image with this height. The blue box is the 3-D bounding box for cell detection.

#### 2.2.2. Neuron Contact Detection Based on Multi-Task Convolutional Network

**Figure 7:**
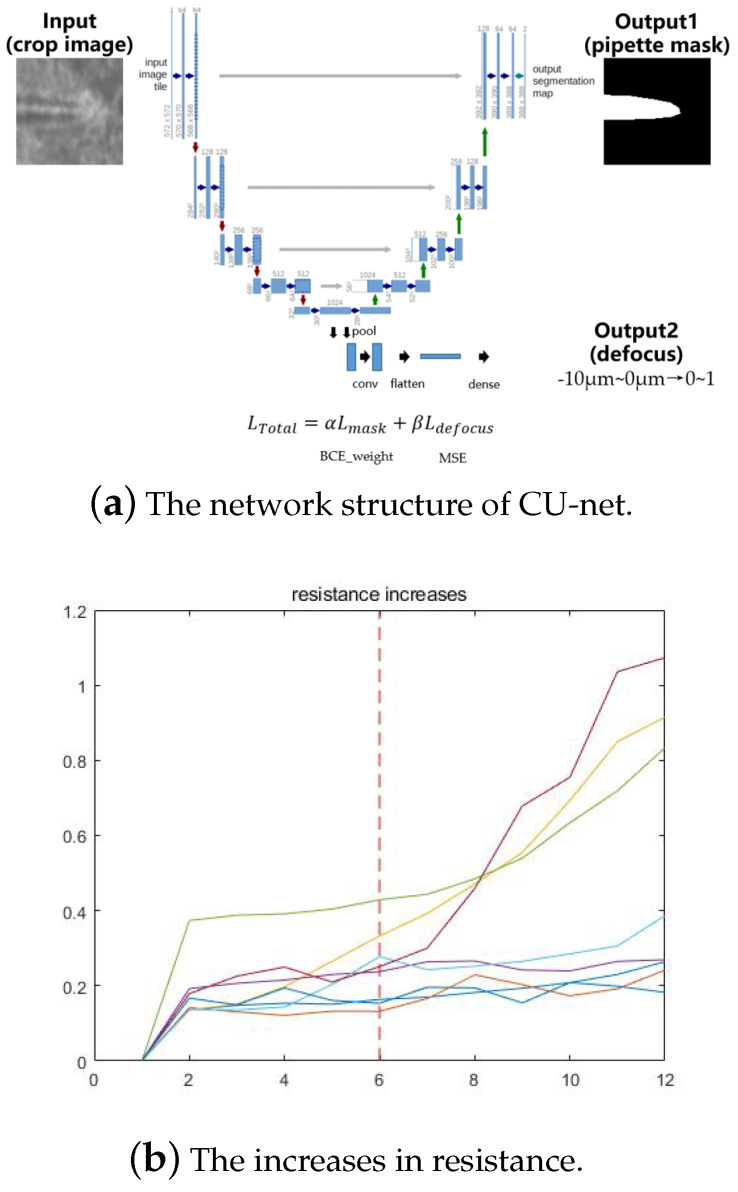
Deep learning-based pipette contact cell detection.

During pipette descent, its movement leads to changes in the brain-slice environment so we cannot use the focusing method in Section 2.1.1 to determine whether the pipette is focused. We propose a neural network called Contact-U-net (CU-net), which is an improvement of U-net. As shown in Figure 7b, the network is simultaneously trained with one single input and multiple outputs. The input of the network is one ROI image with the defocused pipette. The first output is the binary mask image of the pipette, and the second output is the defocus degree of the pipette tip, mapping the defocus amount from 0 to 10 μm to a value between 0 and 1. To obtain the second output, we add a series of layers, including a convolutional layer, a pooling layer, and a fully connected layer, to the last layer of the original U-net encoder. These two tasks share the convolutional module in the encoder part of the network.

In the loss function, we use the binary cross entropy (BCE) as the loss of the segmentation task and the mean squared error as the loss of the defocus calculation task. The total loss of the network is the weighted sum of two losses, with an α of 0.5 and a β of 1 during network training.

(4) $$L_{Total}=\alpha L_{mask}+\beta L_{defocus}$$

In traditional automatic patch-clamp experiments, only a single resistance is used for neuron contact detection. In this study, we determine the detection time of the proposed CU-net using the pipette resistance, which improves the experiment process. We record the pipette resistance from the electrode entering the water environment to coming into contact with the neurons, as shown in Figure 7b. The different colored lines represent the experimental results of different cells. The increase in the resistance value is proportional to the falling distance of the pipette. The red dotted line is selected based on the experienced experimenter, judging the pipette descent height when it comes into contact with the cell based on the image. In 37.5% of experiments, the pipette resistance continues to increase after contact with the neuron, whereas in the other 62.5% of experiments, the resistance value tends to stabilize. At the contact moment, the increases of all the resistance values are less than 0.5 MΩ. The measured value of the resistance values is in steps of 0.1 MΩ, so we set 0.1 MΩ–0.5 MΩ as the start and end times of the CU-net detection.

### 2.3. Automatic Process for Neuron Contact Detection

**Figure 8:**
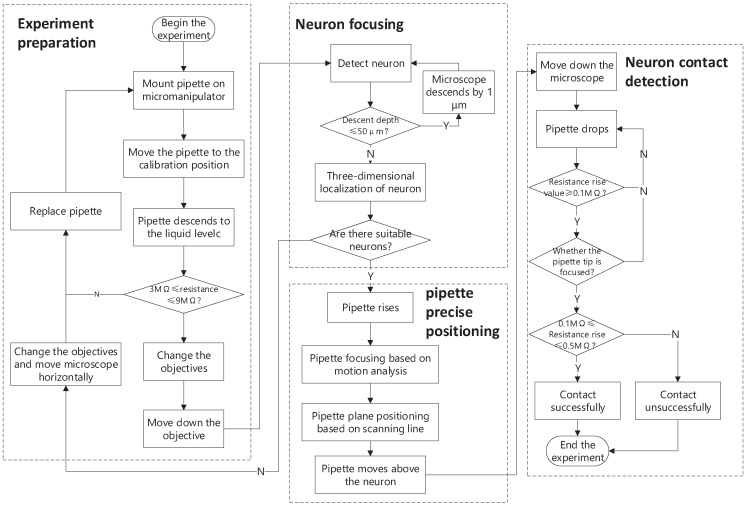
The automatic process for neuron contact detection.

Figure 8 shows the automatic process for neuron contact detection.

1. System initialization under 4× objective lens: It is impossible to ensure that the pipette directly appears in the field of view under the 40× objective lens due to the different shapes of the pipettes and manual assembling. We start the experiment under the 4× objective lens and manually adjust the position of the pipette to appear in the field of view. We move the pipette tip to the calibration position using the PID control algorithm. The calibration position ensures that the pipette tip appears in the field of view under the 40× objective lens. Afterward, we lower the pipette into the artificial cerebrospinal fluid (ACSF) solution and measure the pipette resistance. If the resistance exceeds the specified range, we apply a positive pressure of 10 s to blow away the impurity that blocks the pipette. If the resistance value still cannot reach the specified range, we should replace the pipette for the next experiment.

2. Neuron positioning: After system initialization, we switch the 40× objective lens and lower the objective lens to search for the neurons. The pipette is simultaneously lowered to prevent collision between the pipette and the microscope. We move to the cortex of the brain slice and detect the neurons within a depth range of 50 μm, as described in Section 2.2.1.

3. Pipette precise positioning: We first raise the objective lens of the microscope for pipette searching. During the microscope movement, we the MOG method to detect the motion of the focus region of the pipette. The moment when the focus region is detected is the moment when the pipette changes from the negative defocus to the positive defocus state. The pipette is focused at this moment. For pipette-tip positioning, we combine the object detection model and the scanning line algorithm to obtain the precise position of the tip in its ROI images. Finally, the pipette tip is moved to the place above the selected neuron using the PID algorithm.

4. Neuron contact detection: We move the objective lens of the microscope down to the focal plane of the neuron and then lower the pipette. When the pipette is focused, the pipette has made slight contact with the neuron. Furthermore, the proposed CU-net is used to determine the focus of the pipette tip once the resistance value of the pipette is greater than 0.1 MΩ that of the pipette when it has just entered the ACSF solution. When the defocus degree of the CU-net output is less than 0.2, it is determined that the pipette tip is exactly in the focus plane, that is, the pipette tip has successfully contacted the neuron.

## 3. Experimental Results

### 3.1. Automatic Patch-Clamp System

**Figure 9:**
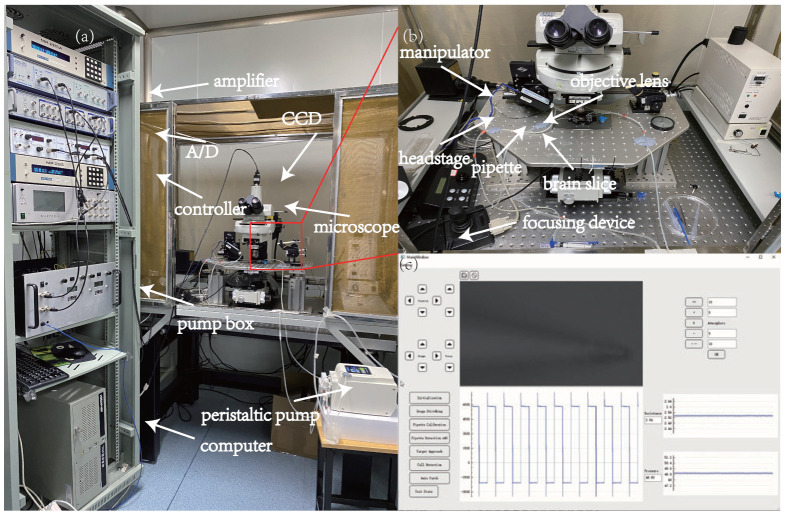
Robotic patch-clamp system. (**a**,**b**) System setup. (**c**) Human–machine interface.

The robotic patch-clamp system was developed within our laboratory [21]. As shown in Figure 9a,b, the upright microscope (Eclipse FN1, Nikon, Tokyo, Japan) is capable of visualizing neurons within a brain slice by moving the stage in the X−Y plane with a repeatability of ±0.01μm and working space of 20 mm × 20 mm (MP285, Sutter Instrument, Sacramento, CA, USA)). A motorized focusing device with a repeatability of ±0.1μm (ES10ZE, Prior, Cambridge, UK) controlled the movement of the microscope in the *Z*-direction to focus the target cell vertically. A micromanipulator with a repeatability of ±0.04μm and a working space of 50 mm × 50 mm × 50 mm (MP285, Sutter Instrument) controlled the 3D movement of the electrode pipette. A CCD camera (IR-2000, DAGE-MTI) was mounted on the microscope to acquire images at 60 fps for image detection and processing during the experiment. A signal amplifier (Multiclamp 700B, Axon Instruments, San Jose, CA, USA) and a data acquisition device (DAQ USB-6211, National Instruments, Austin, TX, USA) were used for electrophysiological data acquisition. An in-house-developed pneumatic pump provided a pressure that could be freely switched from −5 psi to 15 psi with a resolution of 10 Pa for patch-clamp operation. The whole system was covered by an electromagnetic shield to isolate the electric disturbances from the environment.

A host computer was used for microscopic image processing, electric signal acquisition, aspiration pressure control, and motion control for the microscope and manipulators. All of these operations were automated through an in-house-developed framework with a human–machine interface (HMI) written in C++ and QT (see Figure 9c). The experimenter can perform each step of the patch-clamp experiment through the HMI and obtain image information and electrophysiological signal information in real time.

### 3.2. Experimental Results of Pipette Precise Positioning

#### 3.2.1. Experimental Results of Pipette Focusing

**Figure 10:**
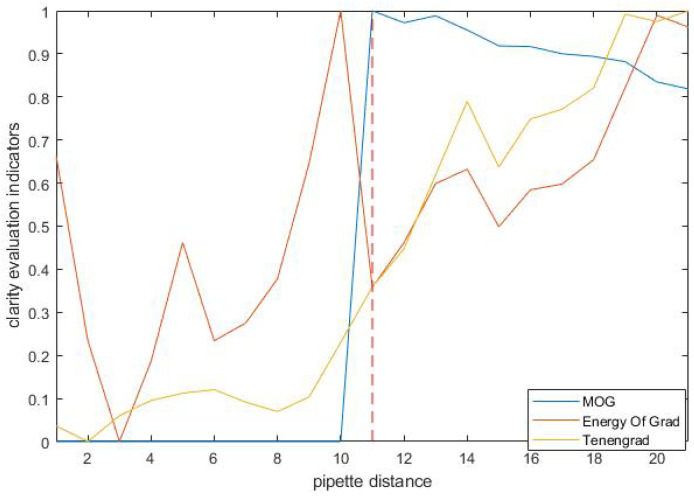
Clarity evaluation curves for three methods.

Since the patch-clamp pipette was mounted on the experimental setup at an oblique angle, the tip focusing height could not be judged using ordinary clarity evaluation functions. In this paper, the method of motion detection was used to detect the focusing height of the needle tip. We used the Energy of Gradient and Tenengrad, two common sharpness evaluation indexes, and the MOG motion detection algorithm to compare the focus positioning of the pipette needle tips, and the results are shown in Figure 10. The red dash line is where the pipette tip is focused. The results of all three methods were processed using normalization, and the red dotted line in the figure is the focus position of the pipette tip. From the results, it can be seen that the clarity evaluation index was in an irregular state, and there was no turning point at the focus. The result of the MOG algorithm is indicated by the blue line, and its physical meaning represents the X-coordinate of the moving pixel detected by the algorithm. In the experiment, the microscope first focused below the height of the pipette and was in negative defocus. After that, the microscope was raised and stopped moving when the method detected that the pipette tip was focused. Since the microscope moved upward, the focal plane moved from the tip to the left to reduce the coordinates of the X-coordinate, so the indicator decreased after focusing on the results. The overall trend of the MOG algorithm is similar to a step function, with 0 before the tip focusing and approximately 1 after focusing.

For the two algorithms of motion detection, MOG and MHI, we detected the movement of 10 groups of pipettes in different brightness environments to obtain the focal plane position of the tip. The MHI algorithm selected two thresholds for comparison, which were used to determine the difference in motion between two frames of pipette movement.

**Figure 11:**
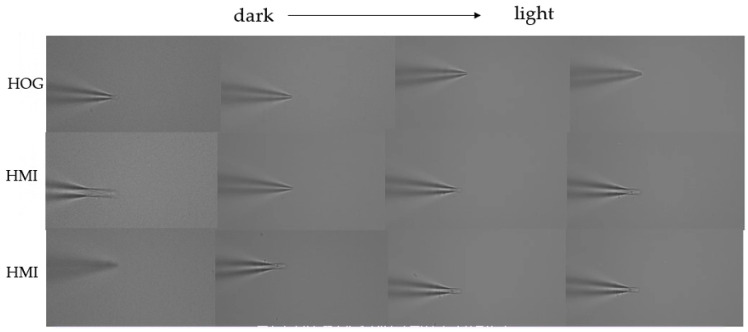
Comparison of the results of the MOG and MHI algorithms.

The comparison results of one of the three methods are shown in Figure 11. The brightness of the experiment increases from left to right. The first line is the experimental result of the MOG algorithm, and the second and third lines are the experimental results of the MHI algorithm using different thresholds. In four different brightness environments, the microscope focused on the tip position of the pipette using the MOG algorithm, as the algorithm can adaptively adjust the parameters of the background model so that different brightness levels yield the same processing results. In the MHI algorithm, which selected different thresholds, the microscope stopped to the left of the tip, which means that the shape of the image could only be detected by the MHI algorithm when the amount of change exceeded the negative defocus state and became the positive defocus state. At different brightness levels, the microscope stopped at different focal planes, indicating that the MHI detection results have a good relationship with the brightness of the experimental environment. In dark conditions, the microscope could not be stopped, indicating that the detection failed. Therefore, the use of MHI motion detection algorithms is not suitable for detecting the height of the pipette tip.

In addition, the difference between the focal plane depth detected by the MOG algorithm and the manually selected focal plane depth at different brightness levels was less than 3 μm, whereas the difference detected by the MHI algorithm was, on average, 9.8 μm. The detection accuracy of MOG was 60% higher than that of MHI, and it was not limited by thresholds. In this paper, the MOG motion detection algorithm was used to locate the tip of the pipette in the depth direction.

#### 3.2.2. Experimental Results of Pipette Plane Positioning

**Figure 12:**
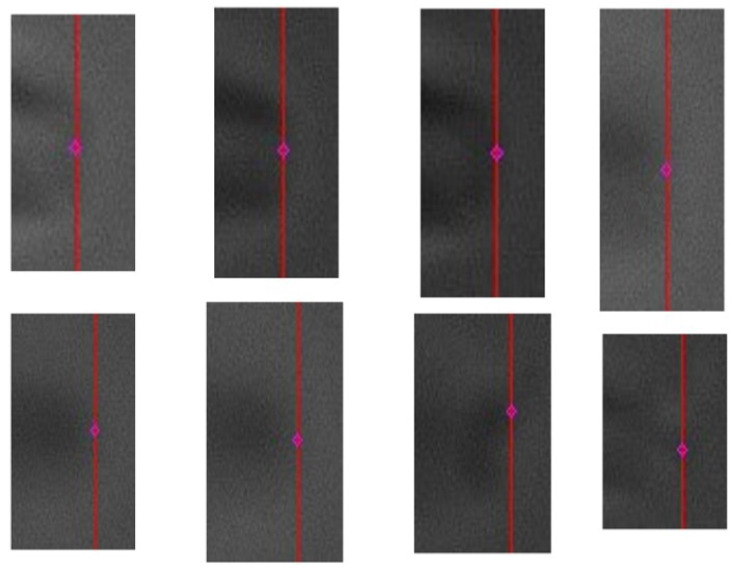
Pipette plane positioning results.

We used the method proposed in this article to detect multiple pipette images with different brightness levels, and the results are shown in Figure 12. The experimental results show that this method has good robustness because the object detection model could detect the bounding box of the pipette at different brightness levels. In the bounding box, the area where the pipette was located was very different from the background area on the right in terms of pixels, so the scanning line algorithm used this difference to determine the coordinate position of the pipette tip. We compared the center point of the bounding box and the detection point of the scanning line algorithm with the appropriate pipette coordinates selected by the laboratory professionals, and the experimental results are shown in Table 1. The results show that the accuracy of the scanning line algorithm was more than 75% higher than that of the simple object detection algorithm and that it can prevent additional errors caused by the production of the target detection model dataset.

### 3.3. Contact Detection Results between Pipette and Neuron in Brain Slice

#### 3.3.1. Maximum Focal Plane Depth Localization of Neuron

**Figure 13:**
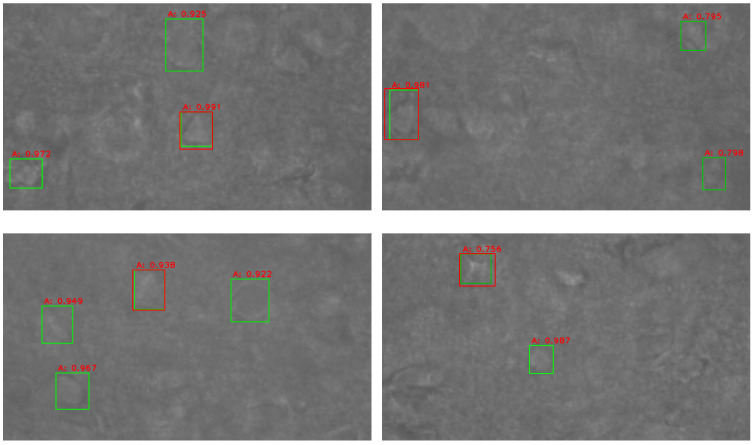
Results of three-dimensional detection of neuronal cells.

In the experimental procedure, the microscope first focused on the plane with the largest area of the neuron. Therefore, we verified the rationality of choosing the middle height through experiments. We performed experiments using brain slices from different mice, and the results are shown in Figure 13. The green bounding box is the result of the object detection model, and the red bounding box is the flat detection result of the three-digit bounding box. In the bounding box fusion step, we took the union of the bounding boxes, so the red bounding box has a larger range than the green bounding box. The image shown in the picture is the middle height of the bounding box. The area of the red bounding box increased by 15.8% compared to the green bounding box, whereas the areas of the bounding boxes detected at other heights exceeded 20% of the area of the middle-height cell bounding box. The results show that when the microscope focused on the plane of the height of the middle height of the three-dimensional bounding box, the neurons in the field of view have the largest focusing area.

#### 3.3.2. Experimental Results of Neuron Contact Detection

**Figure 14:**
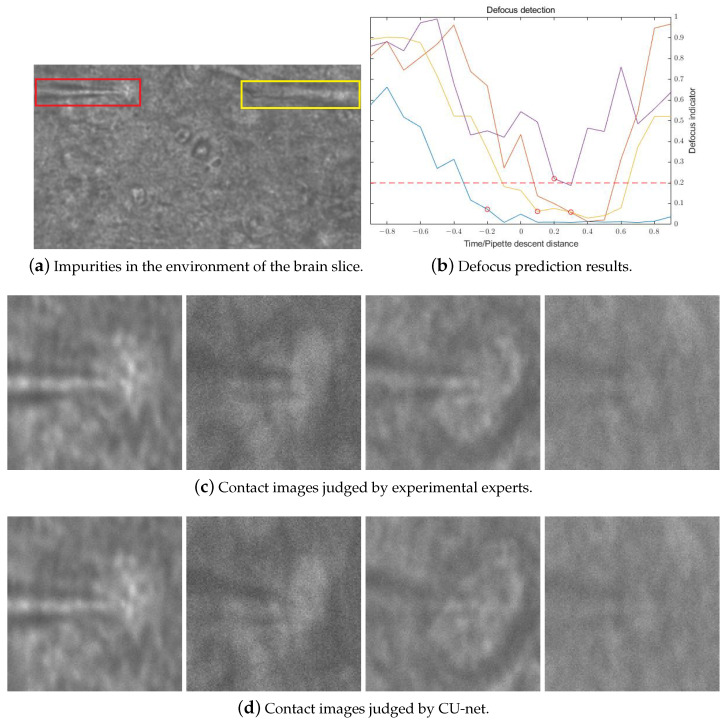
Pipette tip contact cell detection results based on deep learning.

In the dataset of the CU-net network, 11 pictures from the pipette focusing on the cell to the pipette being defocused from 10 μm were selected in steps of 1 μm to simulate the process of the pipette moving down from just above the cell to contact the cell, and the defocusing offset was normalized to 0∼1. In the dataset, the experimental image needed to be cropped to remove impurity noise pixels from the brain-piece environment (Figure 14a). Taking the center point of the cell as the center, we selected 256 pixels to the left to extract more pipette image information and 128 pixels to the right, up and down. Due to memory limitations, the obtained 384 × 256 image resize was resized to 128 × 128 before being sent to the network for training. The output binary image can bias the information extracted by the network more toward the pixels of the pipette, and the network predicts the amount of defocus at this time based on the pixel and shape of the pipette.

When the increase in the resistance of the pipette was in the range [0.1, 0.5] MΩ in the patch-clamp experiment, we used CU-net to determine whether the pipette was focused. The results of the CU-net detection of pipette tips are shown in Figure 14b. The different colored lines represent the experimental results of different cells. We performed patch-clamp experiments using four different mouse brain slices for testing. The dataset contained only negative defocus images, so the index on the left side of the figure shows a linear downward trend, and the positive defocus image on the right side also increased as the tip of the pipette gradually disappeared. The red circle in Figure 14b is the height at which the pipette was in contact with the cell, as chosen by the patch-clamp experimenter. In the three sets of data, the focus position was the result of the linear decline of the indicator and was in the [0, 0.2] interval. Another set of data was also very close to this interval, so we set 0.2 as the threshold to determine whether the tip of the pipette was in contact with the neuronal cell. Figure 14c shows an image of the position of the pipette used to manually judge the contact between the pipette and the cell. Figure 14d shows a location image that used the CU-net network and thresholds to determine whether the pipette was in contact with the cell. The position detected by this threshold was less than 2 μm compared to the actual focus position, which was much smaller than the height of the cell. Our experimental results were manually judged by patch-clamp experts, consistent with the contact between the tip of the pipette and neuronal cells. Therefore, it can be demonstrated that the method proposed in this paper can automate the detection of pipette tips and neuronal cells and improve the success rate of this step in patch-clamp experiments.

## 4. Conclusions

In this paper, we combine image detection methods and electrical resistance analysis to detect the contact between pipette tips and neuronal cells, thereby improving the success rate of automated patch-clamp experiments. This method has two key steps: (1) the pipette moves accurately above the target neuronal cell, aligning the electrode tip with the target cell on a flat surface, and (2) the pipette keeps moving down close to the target neuronal cell until contact is confirmed. In pipette detection, we use the MOG algorithm to detect the movement of the microscope focal plane on the pipette, which does not detect motion when the focal plane is below the pipette and detects motion above the pipette. The moving pixels are approximated by the step function in the results, and the focal height of the tip of the pipette can be determined using the position of its mutation to obtain a clear image of the tip. Compared to the traditional motion detection model, MHI, the MOG algorithm is not affected by the brightness of the image and exhibits good robustness in different environments. The error can be controlled at 3 μm, which ensures the detection of the focusing plane of the pipette tip. In plane detection, we first use the object detection model to obtain the bounding box of the pipette tip, which reduces the range that needs to be detected. Then, the xy pixel position of the needle tip is obtained using the surface line algorithm, and the experimental results show that the error can be controlled within 0.5 μm, a margin significantly smaller than the size of neuronal cells. For neuronal cell detection, we use the object detection algorithm to obtain the bounding box of the cell screen and incorporate the image of its adjacent height to fuse the bounding frames for neuronal cell detection based on the image information of the brain slice at adjacent heights. Finally, we propose a network CU-net that detects whether the pipette tip is focused in the brain-slice environment, which can output the focus of the pipette tip in the brain-slice environment. Further according to the change in resistance of the pipette during contact with the cell, when the rise of the pipette tip compared to the resistance of the initial ACSF is in the range of [0.2, 0.5], we use the network to start the detection. The experimental results show that the difference between the contact height detected by CU-net and the height judged by the patch-clamp experimental experts is less than 2 μm, which is much smaller than the height of a single neuronal cell. Therefore, it can be inferred that the method proposed in this paper can be applied to the contact detection between the tip of the pipette and the neuronal cell.

In future work, we will optimize resistor acquisition using an adaptive derivative estimator and interval observers [29,30]. These methods reduce the measured resistance value to minimize spikes, noise, and other undesirable signals. This prevents sudden resistance jumps from affecting experiments. 

## Figures and Tables

**Table 1:** The vertical scanning line on the pipette tip and the red points on the scanning line represent the pixels whose grayscale values are less than 80% of the reference value.

Bbox Centre	Scanning Line Centre
(1.58, 1.36)	(0.38, 0.24)

## Data Availability

The raw data supporting the conclusions of this article will be made available by the authors, without undue reservation.
